# Health and social care service utilisation and associated expenditure among community-dwelling older adults with depressive symptoms

**DOI:** 10.1017/S2045796020001122

**Published:** 2021-02-02

**Authors:** Shiyu Lu, Tianyin Liu, Gloria H. Y. Wong, Dara K. Y. Leung, Lesley C. Y. Sze, Wai-Wai Kwok, Martin Knapp, Vivian W. Q. Lou, Samson Tse, Siu-Man Ng, Paul W. C. Wong, Jennifer Y. M. Tang, Terry Y. S. Lum

**Affiliations:** 1 Sau Po Centre on Ageing, The University of Hong Kong, Hong Kong, SAR, China; 2 Department of Social Work and Social Administration, The University of Hong Kong, Hong Kong, SAR, China; 3 Care Policy and Evaluation Centre (CPEC), London School of Economics and Political Science, London, UK; 4 School for Social Care Research, National Institute for Health Research, London, UK

**Keywords:** Depression, elderly, health economics, health service research

## Abstract

**Aims:**

Late-life depression has substantial impacts on individuals, families and society. Knowledge gaps remain in estimating the economic impacts associated with late-life depression by symptom severity, which has implications for resource prioritisation and research design (such as in modelling). This study examined the incremental health and social care expenditure of depressive symptoms by severity.

**Methods:**

We analysed data collected from 2707 older adults aged 60 years and over in Hong Kong. The Patient Health Questionnaire-9 (PHQ-9) and the Client Service Receipt Inventory were used, respectively, to measure depressive symptoms and service utilisation as a basis for calculating care expenditure. Two-part models were used to estimate the incremental expenditure associated with symptom severity over 1 year.

**Results:**

The average PHQ-9 score was 6.3 (standard deviation, s.d. = 4.0). The percentages of respondents with mild, moderate and moderately severe symptoms and non-depressed were 51.8%, 13.5%, 3.7% and 31.0%, respectively. Overall, the moderately severe group generated the largest average incremental expenditure (US$5886; 95% CI 1126–10 647 or a 272% increase), followed by the mild group (US$3849; 95% CI 2520–5177 or a 176% increase) and the moderate group (US$1843; 95% CI 854–2831, or 85% increase). Non-psychiatric healthcare was the main cost component in a mild symptom group, after controlling for other chronic conditions and covariates. The average incremental association between PHQ-9 score and overall care expenditure peaked at PHQ-9 score of 4 (US$691; 95% CI 444–939), then gradually fell to negative between scores of 12 (US$ - 35; 95% CI - 530 to 460) and 19 (US$ -171; 95% CI - 417 to 76) and soared to positive and rebounded at the score of 23 (US$601; 95% CI -1652 to 2854).

**Conclusions:**

The association between depressive symptoms and care expenditure is stronger among older adults with mild and moderately severe symptoms. Older adults with the same symptom severity have different care utilisation and expenditure patterns. Non-psychiatric healthcare is the major cost element. These findings inform ways to optimise policy efforts to improve the financial sustainability of health and long-term care systems, including the involvement of primary care physicians and other geriatric healthcare providers in preventing and treating depression among older adults and related budgeting and accounting issues across services.

## Introduction

Financing healthcare and long-term care for older persons is a major challenge for societies facing rapid population ageing (World Health Organization, 2015). Governments should identify and manage costs falling on health and long-term care systems to ensure financial sustainability. Some costs, such as those associated with depression, are potentially avoidable (Schoevers *et al*., 2006).

Depression is a common mental health condition among older adults (World Health Organization, 2017). It often aggravates physical health problems (Porensky *et al*., 2009) and increases healthcare costs (Bock *et al*., 2017). Studies found that older adults with depression generated higher healthcare costs than their non-depressed peers (Lum *et al*., 2013; Ludvigsson *et al*., 2018; König *et al*., 2019). However, economic evidence on impacts of depressive symptom severity on care utilisation and expenditure remains inconclusive because previous studies employed heterogeneous ways to categorise patients (Vasiliadis *et al*., 2013; Bock *et al*., 2016). Studies that used categorical diagnostic instruments (e.g. ICD-10) or cut-off points from dimensional instruments (e.g. Patient Health Questionnaire (PHQ-9)) also failed to examine the gradual increase of healthcare utilisation and expenditure by depression scores.

Additionally, few studies have explored differential impacts of depressive symptom severity on expenditures across care settings as earlier studies mainly focused on healthcare costs (König *et al*., 2019). The uneven distribution of economic evidence across care settings hinders our ability to inform strategic policies to optimise financial resources across settings (Knapp and Wong, 2020).

Evidence also remains sketchy regarding care expenditure associated with depressive symptoms beyond Western countries (Chin *et al*., 2015). East Asian countries have a large and growing ageing population, with the prevalence of late-life depression comparable to Western countries (Guerra *et al*., 2016). Economic evidence generated from Western studies may not be generalised to this region, due to differences in care systems and help-seeking behaviours. Association between care cost and depression across care settings in non-Western countries should be explored.

As in many other East Asian societies, attention to depression is inadequate in Hong Kong (Lam *et al*., 2015), even though over 10% of older adults in primary care settings show signs of clinically significant symptoms (Sun *et al*., 2012). Hong Kong Chinese older adults tend to report more somatic symptoms than their western peers (Chan *et al*., 2015). As public healthcare is virtually free (Ramesh and Holliday, 2001), older adults with depressive symptoms tend to delay appropriate prevention but overuse healthcare (Wong *et al*., 2009).

This study aims to investigate the effects of depressive symptom severity on care utilisation and expenditures across three care settings (healthcare, rehabilitation and social care) among community-dwelling older adults in Hong Kong, using two approaches to define severity: by standard cut-off points and gradual change of PHQ-9 score, respectively. Based on prior studies, we hypothesised that (1) compared to those without depressive symptoms, older adults with higher depressive symptom severity are more likely to use healthcare, rehabilitation and social care services, and have a greater amount of care expenditures and (2) the gradual increase in PHQ-9 score is associated with an incremental increase in care expenditures in all three care settings.

## Methods

### Sample

The current study applied a cross-sectional design. Study data were derived from assessments of 2707 community-dwelling Chinese older adults in a community-based program collaborative stepped-care service for preventing late-life depression implemented in Hong Kong between September 2016 and September 2019. To be eligible, respondents needed to (1) be aged 60 years or older; (2) have mild or above depressive symptoms (scoring ⩾5 on PHQ-9), or PHQ-9 < 5 and at least one of the following risk factors for elderly depression, identified by existing literature (Schoevers *et al*., 2006; Sun *et al*., 2012), including frequently feeling lonely, lack of social interaction, lack of meaningful/enjoyable activities, chronic pain, more than four chronic diseases, bereavement and previous diagnosis of depression/anxiety. Those with a known history of autism, intellectual disability, schizophrenia-spectrum disorder, bipolar disorder, Parkinson's disease or dementia were excluded. Those with significant suicidal risk, assessed by trained social workers using the self-harm inventory (Gratz, 2001), were excluded and referred to professional care. All data were collected by trained social workers through face-to-face interviews.

### Service utilisation and expenditure

We focus on service utilisation and expenditure in three care settings: healthcare (inpatient/outpatient psychiatric care and non-psychiatric care, community-based psychiatrists/psychiatric nurses, community-based general practitioners and nurses), rehabilitation (day-hospital, physiotherapist and occupational therapist consultation) and social care (professional social work services, clinical psychologist services and non-professional community support services such as chore services and meal delivery). We categorised clinical psychologist services into social care sector as clinical psychologists are required as part of a multi-disciplinary professional team in mental health-related social service (The Hong Kong Council of Social Service, 2015). Trained social workers collected information on respondents' service utilisation for the previous 3 months during face-to-face interviews using the locally adapted Client Service Receipt Inventory (CSRI) (Beecham and Knapp, 1992). A team of experienced researchers with strong health services research and health economics backgrounds adapted the CSRI and piloted it with a small sample of older persons in 2016. We calculated 3-month and then annual expenditure by multiplying the volume of service usage and unit cost of these services obtained from the government (2017/2018 prices). All costs were converted to US dollars using the official exchange rate. Service categories and unit costs are shown in online Supplementary Table 1.

### Depressive symptom severity

Depressive symptom severity was assessed using the Chinese version of the PHQ-9 (Yeung *et al*., 2008). PHQ-9 scores range from 0 to 27, with cut-off points of 5, 10 and 15 suggesting non-depressed (PHQ-9 = 0–4), mild (PHQ-9 = 5–9), moderate (PHQ-9 = 10–14) and moderately severe (PHQ-9 ⩾ 15) symptoms, respectively (Kroenke *et al*., 2000; Chen *et al*., 2010). Cronbach alpha in this sample was 0.71, showing good internal consistency.

### Covariates

Control variables included gender (female *versus* male (the reference group)), age (years), marital status (married versus divorced/single/widowed (the reference group)), years of education, poverty status (recipient of means-tested welfare payment versus non-recipient (the reference group)) and living arrangements (living alone versus living with others (the reference group)). The common chronic condition was assessed by a single question about whether participants have over four common chronic conditions (yes versus no (the reference group)). Cognition was measured using the Hong Kong Chinese version of the Montreal Cognitive Assessment 5-minute protocol (MoCA) (Wong *et al*., 2015). These covariates were included as suggested in the literature (Garrido *et al*., 2011). We also controlled for a previous diagnosis of depression/anxiety (yes versus no (the reference group)). A sensitivity analysis was performed to check the robustness of results without controlling for a previous diagnosis of depression/anxiety.

### Data analyses

Respondents were grouped according to their PHQ-9 scores into one of four groups: non-depressed, mild, moderate and moderately severe symptoms. We used Chi-square or one-way ANOVA to examine between-group differences in respondents' characteristics. We used logistic regression to examine effects of depressive symptoms on service utilisation across care settings and used the generalised linear model (GLM) with the log link and gamma distribution to estimate the effects on expenditure among those who used services as expenditure data were highly skewed (Deb and Norton, 2018). In both parts of the model, we controlled for gender, age, marital status, education, chronic condition, cognitive function, living arrangements and poverty status. We estimated predicted expenditure 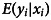 across the four groups (*x*_*i*_) for each care setting using the following formula:1
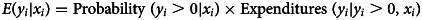


The formula (1) indicates that the expected expenditure is the product of the probability of having a positive expenditure and the amount of care expenditures when participants use any service. This two-part model (TPM) was preferred because some respondents did not use any health/rehabilitation/social care services during the previous 3 months (Belotti *et al*., 2015). We ran TPMs to estimate the incremental effect of severity of depressive symptoms on expenditures across three care settings. Incremental expenditure associated with a severity level of depressive symptoms for a respondent is the difference in predicted expenditure with and without depressive symptoms for that respondent (Basu and Rathouz, 2005). The incremental/marginal effect (average marginal effect (AME)) for the severe symptoms of interest, *d_i_*, can be written as:2
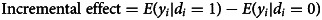


We used the *twopm* command for running TPM analysis (Belotti *et al*., 2015) and obtained the AMEs using the *margins* command (Williams, 2012; Belotti *et al*., 2015) in Stata V15.1. Estimates of AMEs were bootstrapped 5000 times to obtain confident interval (CI). We first tested the effects of severity symptoms using cut-off points and then repeated our TPM analysis with PHQ-9 score as a *continuous* variable. Noting potential non-linear relationships between PHQ-9 score and costs, we first ran the curve estimation regression statistics (linear, quadratic and cubic) and then decided whether to include squared and cubic terms of PHQ-9 score in the TPM. The *marginsplot* command was used to capture the AMEs of the discrete change in PHQ-9 score on costs (Williams, 2012).

## Results

Table 1 presents respondents' demographic and socio-economic characteristics. Most were female (78.2%), 39.2% were married, 40.8% lived alone and 29.0% received means-tested welfare benefits, average age was 76.4 years (s.d. = 8.3) and average formal education was 4.9 years (s.d. = 4.6). Average MoCA score was 21.6, and 11.1% had four or more chronic diseases and 8.5% had previously been diagnosed with depression or anxiety. Average PHQ-9 score was 6.3 (s.d. = 4.0) and 31.0% were non-depressed, 51.8% had mild depressive symptoms, 13.5% had moderate depressive symptoms and 3.7% had moderately severe depressive symptoms. Significant between-group differences were evident only as regards living arrangements PHQ-9 score and prior diagnosis of depression or anxiety.

**Table 1.** Characteristics of study respondents (*N* = 2707)

	ALL (*n* = 2707)	Non-depressed (*n* = 838, 31.0%)	Mild (*n* = 1,403, 51.8%)	Moderate (*n* = 367, 13.5%)	Moderately severe (*n* = 99, 3.7%)	*p value*
	*N* (%)/Mean (s.d.)	*N* (%)/Mean (s.d.)	*N* (%)/Mean (s.d.)	*N* (%)/Mean (s.d.)	*N* (%)/Mean (s.d.)	
Female	2118 (78.2)	637 (76.0)	1109 (79.0)	298 (81.2)	74 (74.8)	0.143
Married	1062 (39.2)	318 (38.0)	570 (40.6)	133 (36.2)	41 (41.4)	0.348
Age (60–99 years)	76.4 (8.3)	76.9 (8.3)	76.6 (8.4)	75.7 (8.0)	74 (9.1)	0.186
Education (0–21 years)	4.9 (4.6)	4.8 (4.4)	5.1 (4.6)	4.9 (4.7)	4.5 (4.2)	0.845
Living arrangements						0.042
Alone	1105 (40.8)	358 (42.7)	570 (40.6)	139 (37.9)	38 (38.4)	
With spouse	627 (23.2)	199 (23.8)	340 (24.2)	67 (18.3)	21 (21.2)	
With children	330 (12.2)	105 (12.5)	158 (11.3)	55 (15.0)	12 (12.1)	
With others	645 (23.8)	176 (21.0)	335 (23.9)	106 (28.9)	28 (28.3)	
Poverty status: Welfare recipients	785 (29.0)	238 (28.4)	410 (29.2)	105 (28.6)	32 (32.3)	0.857
MoCA (range 9.5–30)	21.6 (4.4)	21.7 (4.5)	21.5 (4.3)	21.7 (4.4)	21 (4.4)	0.492
With more than four chronic diseases	301 (11.1)	99 (11.8)	150 (10.7)	37 (10.1)	15 (15.2)	0.417
Previous diagnosis of depression/anxiety	231 (8.5)	48 (5.7)	123 (8.8)	39 (10.6)	21 (21.2)	<0.001
PHQ-9 (range 0–23)	6.3 (4.0)	2.2 (1.5)	6.7 (1.4)	11.6 (1.5)	17.4 (2.2)	<0.001

Notes: *p* values are calculated using Chi-square/ANOVA test. MoCA, Montreal Cognitive Assessment; PHQ-9, Patient Health Questionnaire-9.

Table 2 shows unadjusted service utilisation and annual care expenditure: 9.9% used psychiatric healthcare, 82.7% used non-psychiatric healthcare and 84.5% used healthcare services in total; 5.3% used rehabilitation services and 12.9% used social care. Overall, 86.3% of the respondents used at least one type of care service. The numbers of respondents who did not use healthcare, rehabilitation and social care services were 419 (15.5%), 2563 (94.7%) and 2359 (87.1%), respectively. There were significant associations between service utilisation and symptom severity. Average psychiatric care expenditure was US$148 (s.d. = US$969), compared with US$4260 (s.d. = US$27 551) for non-psychiatric healthcare. Average rehabilitation expenditure was US$215 (s.d. = US$1846) and social care expenditure was US$91 (s.d. = US$1817). Thus, the average unadjusted annual total care expenditure among all respondents was US$4714 (s.d. = US$27 796). There were significant differences in psychiatric and non-psychiatric care expenditure across groups. Overall, the mild group generated the highest overall care expenditure (US$6346) (s.d. = US$37 146), followed by the moderately severe group (US$5689) (s.d. = US$12 622).

**Table 2.** Percentage of care utilisation and unadjusted annual care expenditures (US$) by care settings (*N* = 2707)

	Health Care (A)			Rehabilitation (B)	Social care (C)	Total (A + B + C)
	Psychiatric care	Non-psychiatric care	Subtotal			
Care utilisation, %						
All respondents	9.9	82.7	84.5	5.3	12.9	86.3
By severity level						
Non-depressed	5.0	78.8	79.8	3.2	13.4	82.7
Mild	9.2	84.1	86.0	6.1	10.1	87.5
Moderate	18.8	83.9	86.9	5.7	19.1	88.0
Moderately Severe	27.3	91.9	93.9	10.1	24.2	93.9
*Chi-square*	89.62***	17.31**	24.84***	13.76**	33.68***	16.58**
Care expenditures, Mean (s.d.)						
All respondents	148 (969)	4260 (27 551)	4408 (27 561)	215 (1846)	91 (1817)	4714 (27 796)
By severity level						
Non-depressed	60 (321)	2150 (11 157)	2210 (11 162)	112 (1080)	70 (758)	2392 (11 307)
Mild	129 (800)	5795 (36 851)	5924 (36 848)	306 (2377)	117 (2452)	6346 (37 146)
Moderate	369 (1999)	3000 (7875)	3369 (8177)	95 (575)	48 (204)	3511 (8213)
Moderately Severe	344 (812)	5042 (12 563)	5386 (12 541)	244 (1341)	59 (149)	5689 (12 622)
ANOVA test	10.32***	3.38*	3.42*	2.53	0.21	3.84**

Notes: s.d., standard deviation. * *p* < 0.05; ** *p* < 0.01; *** *p* < 0.001.

Table 3 shows the results of the logit regression on service utilisation and GLM analyses on care expenditure among those who used services. Compared with the non-depressed group, respondents in the mild (odd ratio, OR = 1.57, *p* < 0.001), moderate (OR = 1.72, *p* < 0.01) and moderately severe (OR = 4.00, *p* < 0.01) groups reported a higher likelihood of healthcare usage after controlling for chronic diseases, cognition and other covariates. Mild, moderate and moderately severe groups were also more likely to use rehabilitation and social care services. Among those who used healthcare services, the moderately severe group incurred the greatest healthcare expenditure (*β* = 1.22, *p* < 0.001), followed by the mild group (*β* = 0.93, *p* < 0.001). Overall, the moderately severe group were more likely to use any type of care services (OR  =  3.44, *p* < 0.01) and generated the greatest expenditure (*β* = 1.18, *p* < 0.001).

**Table 3.** Results of the two-part model analysis on care expenditures

	Health care		Rehabilitation		Social care		Total	
	Logit	GLM	Logit	GLM	Logit	GLM	Logit	GLM
	OR (s.e.)	*β* (s.e.)	OR (s.e.)	*β* (s.e.)	OR (s.e.)	*β* (s.e.)	OR (s.e.)	*β* (s.e.)
*Severity level (ref.: non-depressed)*								
Mild	1.57***	0.93***	1.98**	0.41	0.75*	0.53	1.49**	0.95***
	(0.18)	(0.14)	(0.45)	(0.34)	(0.10)	(0.27)	(0.18)	(0.14)
Moderate	1.72**	0.58***	1.86*	−0.58	1.70**	−0.40	1.61*	0.54***
	(0.31)	(0.15)	(0.56)	(0.37)	(0.29)	(0.28)	(0.30)	(0.15)
Moderately severe	4.00**	1.22***	2.99**	−0.36	2.29**	−0.08	3.44**	1.18***
	(1.73)	(0.31)	(1.18)	(0.49)	(0.63)	(0.32)	(1.51)	(0.30)
*Control variables*								
Female	0.94	−0.96***	0.93	−0.23	0.86	−0.67*	0.89	−0.93***
	(0.13)	(0.19)	(0.20)	(0.28)	(0.13)	(0.28)	(0.13)	(0.18)
Married	0.91	−0.28	1.01	0.11	1.18	−0.22	0.91	−0.28
	(0.12)	(0.17)	(0.22)	(0.30)	(0.20)	(0.24)	(0.13)	(0.17)
Age	1.03**	0.03***	1.00	−0.02	1.01	−0.02	1.04***	0.02**
	(0.01)	(0.01)	(0.01)	(0.02)	(0.01)	(0.01)	(0.01)	(0.01)
Years of education	1.01	0.00	0.99	−0.01	0.99	0.07*	1.01	0.00
	(0.01)	(0.02)	(0.02)	(0.03)	(0.01)	(0.03)	(0.01)	(0.02)
Living alone	0.97	−0.29	1.06	0.16	2.34***	−0.67**	1.17	−0.29
	(0.14)	(0.20)	(0.22)	(0.29)	(0.37)	(0.24)	(0.17)	(0.19)
Poverty status: Welfare recipients	1.28	0.14	1.07	0.61*	1.62***	1.46***	1.36*	0.21
	(0.17)	(0.16)	(0.20)	(0.27)	(0.21)	(0.26)	(0.20)	(0.16)
MoCA	1.00	0.00	0.99	0.05	1.00	−0.03	1.00	0.00
	(0.02)	(0.02)	(0.02)	(0.04)	(0.02)	(0.03)	(0.02)	(0.02)
Previous diagnosis of depression/anxiety	1.56	0.19	1.39	0.06	1.10	0.08	1.48	0.19
	(0.36)	(0.16)	(0.38)	(0.40)	(0.23)	(0.26)	(0.35)	(0.16)
With more than four chronic diseases	2.18***	0.12	2.80***	0.19	1.27	0.34	2.45***	0.19
	(0.48)	(0.16)	(0.60)	(0.29)	(0.23)	(0.38)	(0.60)	(0.15)
Constant	0.53	6.33***	0.05*	8.26***	0.03***	7.64***	0.27	6.79***
	(0.44)	(0.85)	(0.07)	(1.76)	(0.03)	(1.18)	(0.22)	(0.83)

Notes: MoCA, Montreal Cognitive Assessment; OR, odds ratio; s.e., standard error; *β*, coefficient; GLM, generalised linear model; Standard errors in parentheses. * *p* < 0.05; ** *p* < 0.01; *** *p* < 0.001.

Female respondents generated less healthcare and social care expenditures, and age was associated with a higher likelihood of healthcare usage and expenditure; respondents with more education had greater social care expenditure; welfare recipients were more likely to have social care usage and generated greater rehabilitation and social care expenditures; and those living alone were more likely to use social care; those with more than four chronic diseases were more likely to use healthcare and rehabilitation care. Results of the sensitivity analysis of the TPM that did not adjust for a previous diagnosis of depression/anxiety were almost the same as that of the TPM controlling for a previous diagnosis of depression/anxiety (online Supplementary Table 2).

Table 4 shows predicted expenditures and AMEs across care settings by levels of depressive symptom severity after controlling for covariates. The moderately severe group generated the largest predicted overall care expenditure (US$8104; 95% CI US$7925–US$8284), followed by the mild group (US$6066; 95% CI US$5928–US$6206) and the moderate group (US$4061; 95% CI US$3968–US$4153). The non-depressed group generated the lowest predicted total care expenditure (US$2218; 95% CI US$2166–US$2270). The moderately severe group generated the largest average incremental expenditure (US$5886; 95% CI US$1126–US$10 647 or a 272% increase), followed by the mild group (US$3849; 95% CI US$2520–US$5177, or a 176% increase) and the moderate group (US$1843; 95% CI US$854–US$2831 or 85% increase). All these average marginal associations were significant.

**Table 4.** Incremental associations between depressive symptom severity with health care, rehabilitation and social care (US$) with bootstrapped 95% CI (*N* = 2707)

Severity level	Health care			Rehabilitation care			Social care			Overall		
	Predicted $ (95% CI)	IC $ (95% CI)	% Diff	Predicted $ (95% CI)	IC $ (95% CI)	% Diff	Predicted $ (95% CI)	IC $ (95% CI)	% Diff	Predicted $ (95% CI)	IC $ (95% CI)	% Diff
Non-depressed	2069			108			66			2218		
	(2016–2122)			(105–111)			(62–69)			(2166–2270)		
Mild	5615	3546***	173	310	202*	190	88	22	32	6066	3849***	176
	(5474–5756)	(2277–4815)		(301–320)	(23–382)		(84–92)	(−27 to 72)		(5928–6206)	(2520–5177)	
Moderate	4012	1943***	96	108	1	1	67	1	5	4061	1843***	85
	(3911–4112)	(923–2963)		(105–112)	(−126 to 130)		(64–70)	(−41 to 43)		(3968–4153)	(854–2831)	
Moderately severe	8079	6009*	297	209	101	97	113	48	82	8104	5886*	272
	(7883–8275)	(1025–10 994)		(203–215)	(−208 to 410)		(107–119)	(−41 to 137)		(7925–8284)	(1126–10 647)	

Notes: Results controlled for gender, age, marital status, education, poverty status, living alone, cognitive function, chronic diseases and a history of a diagnosis of depression/anxiety. IC, incremental cost; Diff, difference; CI, confidence interval. CI in parentheses.

* *p* < 0.05; ** *p* < 0.01; *** *p* < 0.001.

Similarly, the moderately severe group generated the highest predicted healthcare expenditure of US$8079 (95% CI US$7883–US$8275), followed by the mild group (US$5615; 95% CI US$5474–US$5756), and moderate group (US$4012; 95% CI US$3911–US$4112). The moderately severe group generated the largest average incremental healthcare costs (US$6009; 95% CI US$1025–US$10 994 or a 297% increase), followed by mild group (US$3546; 95% CI US$2277–US$4815, or 173% increase) and moderate group (US$1943; 95% CI US$923–US$2963 or 96% increase). All average marginal associations were statistically significant. However, the mild group generated the largest and significant average incremental rehabilitation care expenditure (US$202; 95% CI US$23–US$382 or 190% increase), while average marginal associations between symptom severity with social care expenditure were not significant.

Table 5 shows results of TPM analysis of psychiatric and non-psychiatric healthcare utilisation and expenditure. Depressive symptom severity was significantly associated with psychiatric care usage. However, among those who used care, symptom severity was not significantly associated with psychiatric care expenditure. For non-psychiatric care, symptom severity was linked to both likelihoods of non-psychiatric care use and expenditure. Compared with the non-depressed group, the moderately severe group was more likely to use non-psychiatric healthcare (OR = 3.43, *p* < 0.01), followed by the mild (OR = 1.46, *p* < 0.001) and the moderate (OR = 1.48, *p* < 0.05) groups. The moderately severe group generated the greatest predicted expenditure (US$7855; 95% CI US$7658–US$8052), followed by the mild group (US$5502; 95% CI US$5360–US$5645) and the moderate group (US$3570; 95% CI US$3477–US$3662). Average marginal associations of symptom severity on non-psychiatric care were significant.

**Table 5.** Results of two-part model analysis, predicted expenditures and incremental costs of psychiatric and non-psychiatric care (US$) with bootstrapped 95% CI (*N* = 2707)

	Psychiatric care					Non-psychiatric care				
	Logit	GLM	Predicted $	IC $	% Diff	Logit	GLM	Predicted $	IC $	% Diff
	OR (s.e.)	*β* (s.e.)	(95% CI)	(95% CI)		OR (s.e.)	*β* (s.e.)	(95% CI)	(95% CI)	
Severity level (ref.: non-depressed)			77					2011		
			(74–81)					(1958–2065)		
Mild	1.78**	0.02	127	50*	74	1.46***	0.94***	5502	3491***	176
	(0.34)	(0.17)	(122–132)	(6–94)		(0.17)	(0.15)	(5360–5645)	(2219–4764)	
Moderate	4.09***	0.27	321	235***	368	1.48*	0.51**	3570	1558**	79
	(0.93)	(0.18)	(303–321)	(104–365)		(0.25)	(0.16)	(3477–3662)	(598–2518)	
Moderately severe	5.08***	0.07	299	221**	359	3.43**	1.21***	7855	5844*	298
	(1.60)	(0.20)	(291–307)	(65–377)		(1.31)	(0.34)	(7658–8052)	(554–11 135)	
Control variables										
Female	0.84	0.30*				1.04	−1.00***			
	(0.16)	(0.14)				(0.14)	(0.19)			
Married	0.67*	0.08				0.96	−0.25			
	(0.12)	(0.14)				(0.13)	(0.17)			
Age	0.95***	0.00				1.04***	0.03***			
	(0.01)	(0.01)				(0.01)	(0.01)			
Years of education	1.02	0.01				1.02	−0.01			
	(0.02)	(0.02)				(0.01)	(0.02)			
Living alone	0.92	0.42*				0.91	−0.29			
	(0.17)	(0.16)				(0.12)	(0.20)			
Poverty status: Welfare recipients	1.33	0.26				1.16	0.12			
	(0.23)	(0.16)				(0.15)	(0.16)			
MoCA	1.02	−0.02				0.99	0.00			
	(0.02)	(0.02)				(0.01)	(0.02)			
Previous diagnosis of depression/anxiety	9.03***	−0.25*				0.94	0.14			
	(1.63)	(0.13)				(0.18)	(0.18)			
With more than four chronic diseases	0.85	−0.38**				2.37***	0.13			
	(0.19)	(0.13)				(0.51)	(0.16)			
Constant	1.22	7.01***				0.27	6.22***			
	(1.48)	(0.79)				(0.21)	(0.90)			

Notes: MoCA, Montreal Cognitive Assessment; OR, odds ratio; s.e., standard error; *β*, coefficient; GLM, generalised linear model; IC, incremental cost; Diff, difference; CI, confidence interval; s.e. and CI in parentheses. * *p* < 0.05; ** *p* < 0.01; *** *p* < 0.001.

**Fig. 1.**
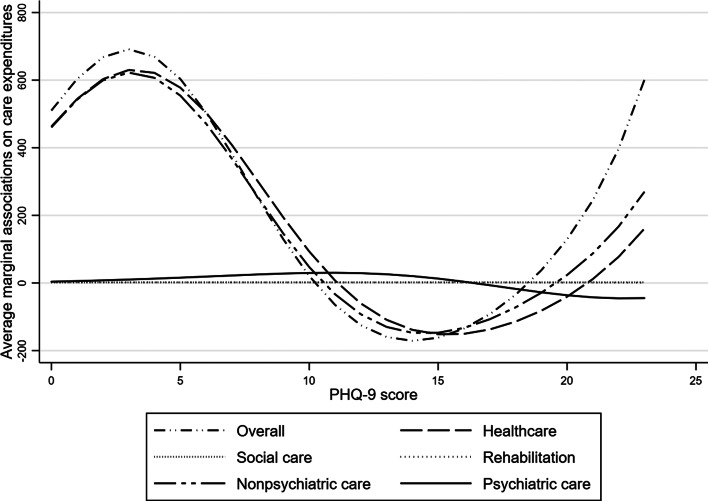
Results of average marginal associations on care expenditures using PHQ-9 score as a continuous variable. Notes: Results controlled for gender, age, marital status, education, poverty status, living alone, cognitive function, chronic diseases and a history of a diagnosis of depression/anxiety. The marginal associations were calculated based on the two-part model analyses in online Supplementary Table 3. TPM, two-part model. The Patient Health Questionnaire-9 (PHQ-9) score ranged from 0 to 23 in this sample.

We repeated TPM analysis using PHQ-9 score as a *continuous* variable. After examining the curve fitness for each care setting, we included squared and cubic terms of PHQ-9 score for healthcare, psychiatric and non-psychiatric care (online Supplementary Table 3). Figure 1 illustrates AMEs of PHQ-9 score for all cost categories. The cubic relationship between PHQ-9 score and the incremental cost was found in non-psychiatric care setting. Healthcare and overall care expenditures followed a similar pattern, as non-psychiatric care was the major cost component. The average incremental association between PHQ-9 score and overall care expenditure peaked at the PHQ-9 score of 4 (US$691; 95% CI US$444–US$939), then gradually fell to negative between 12 (US$-35; 95% CI US$-530 to US$460) and 19 (US$-171; 95% CI US$-417 to US$76) scores and soared to positive and rebounded at the score of 23 (US$601; 95% CI US$-1652 to US$2854). AMEs on psychiatric care fluctuated less than non-psychiatric care while AMEs on social care and rehabilitation were flat.

## Discussion and implications

To our knowledge, this is the first study to systematically estimate increased care costs associated with depressive symptom severity among older adults in Asia and across three care settings. Our findings highlight significant economic impacts on health and social care systems associated with the depressive symptom.

Depressive symptom severity and PHQ-9 scores were significantly associated with care utilisation and expenditure: compared to older adults without depressive symptoms, those with mild, moderate and moderately severe symptoms were more likely to use healthcare, rehabilitation and social care, and had higher care expenditures in healthcare settings, especially non-psychiatric care, which were consistent with previous studies (König *et al*., 2019).

We also captured the complexity of impacts of depressive symptoms on costs. A cubic relationship between symptom severity and care expenditure was found: moderately severe symptoms were associated with the largest and significant increase in healthcare and its subgroup non-psychiatric care, followed by mild symptoms. These incremental associations on care expenditure in the mild-symptom group were substantially greater than for moderate symptoms. Consistent with existing literature (Lum *et al*., 2013), the present study finds evidence of greater care expenditure associated with mild depressive symptom than with moderate symptoms. It is possible that mild level depressive symptoms are mainly presented as somatic symptoms (e.g. appetite change), and thus older adults with mild symptoms may seek help from general care sector, while those with moderate symptoms lack energy to seek help because of their depression (Parker *et al*., 2001).

This pattern persisted when incremental effects of PHQ-9 score on care were examined, providing even finer graduation in associations with care costs. Specifically, inverted U-shaped relationships were found in the healthcare setting when older adults scored 0–9 on PHQ-9, while U-shaped relationships were found among those scoring 10–23 on PHQ-9 in healthcare and non-psychiatric care settings. Such patterns were also found in total care expenditure. Older adults with the same symptom severity can therefore have different care needs and utilisation. Older adults who scored 10 on PHQ-9 generated positive average incremental overall care costs while those scoring 14 had negative average incremental overall care costs. It is possible that older adults with PHQ-9 score of 0–4 are sensitive to emerging symptoms and associate such changes with physical health problems (Lee *et al*., 2007) and thus prefer to seek help in non-psychiatric healthcare settings. In contrast, older adults with moderate symptoms are reluctant to seek help due to stigma (Parker *et al*., 2001). When depressive symptoms become moderately severe, older adults with some alarming symptoms, like suicidal ideation, get noticed by family members who may refer them to treatment (Wong *et al*., 2009). Moderately severe depression in older people may exacerbate disability and symptoms of chronic diseases, hindering their self-care ability, which leads to increase in hospitalisation or visits in Accident and Emergency department (Wong *et al*., 2009).

Therefore, careful attention should be paid to care need (both amount and types) of older adults *within* symptom severity categories. Relevantly, these findings suggest two opportunity windows for timely prevention/treatment. The first lies at the PHQ-9 score of 0–4: even though overall care cost is low when older adults are not depressed, every single unit increase in PHQ-9 is associated with larger incremental cost (US$510–US$691); the second window lies at PHQ-9 score of 15–20 when care expenditure is mounting before marginal effects rebound.

This study found that after controlling for other covariates including comorbid conditions, higher symptom severity was positively and significantly associated with non-psychiatric healthcare expenditure. Having more than four chronic diseases was independently associated with higher likelihood of non-psychiatric care utilisation yet smaller psychiatric care expenditure. These findings seem to suggest that older Asian people are more likely to see depressive symptoms as a somatic illness because the bodily experience is regularly coupled with the expression and experience of emotional distress (Lee *et al*., 2007), leading to a significant increase in non-psychiatric care expenditure. Meanwhile, the findings suggest that depressive symptoms significantly affect the care of other chronic illnesses, leading to a significant increase in non-psychiatric care expenditure. Future research should explore how depressive symptoms interact with specifically identified commodities to affect health/social care use/expenditures.

We also observed that sociodemographic factors including gender and education were significantly associated with health and social care utilisation. Previous evidence related to the gender difference in healthcare utilisation remains mixed (Redondo-Sendino *et al*., 2006; Vegda *et al*., 2009). We found that females used more psychiatric care than males and males used more non-psychiatric care than females. It seems that men were substituting psychiatric care with non-psychiatric care, likely because they were more affected by stigma associated with depression than females (McVittie and Willock, 2006). Our study also found that years of education was only positively and significantly related to social care expenditures, while findings of prior studies on education and care utilisation remained inconclusive (Chao *et al*., 2013).

The findings also underline the effects of living arrangement and poverty status on service use and/or care costs. Consistent with previous studies (König *et al*., 2019), we found that older adults living alone were more likely to utilise social care and had greater psychiatric care expenditures. One explanation is that older adults who are more isolated lack social connection, therefore worsening overall wellbeing. This finding suggests the need to provide additional support to those living alone. Older adults who were welfare recipients were more likely to use rehabilitation and social care. As all mean-tested recipients in Hong Kong are entitled to free healthcare at public hospitals or clinics, they may be inclined to overuse care services.

Our study has both strengths and limitations. Strengths include a large sample, data collection by professionally trained social workers, service use data from three settings, differentiation between psychiatric and non-psychiatric healthcare, adjustment for a comprehensive array of covariates, cost estimation using TPM, levels of depression measurement based on PHQ-9 scores and use of PHQ-9 as a continuous variable to examine non-linear cost-symptoms relationships.

Our limitations include, first, the sample was not chosen randomly: respondents were recruited from social service centres and mental wellness centres for a project serving older adults at risk of, or who exhibited mild-to-moderate depressive symptoms. This group is probably more vulnerable than the general older population. Second, we only estimated direct medical costs, excluding costs associated with medication and informal care, thus underestimating overall economic impacts. Third, healthcare utilisation data were self-reported and possibly subject to recall bias. We used CSRI to collect data over a 3-month retrospective period while PHQ-9 score and other covariates were collected on the last date of that period. Fourth, we used a cross-sectional design which limited our ability to explore causal relationships between depressive symptoms and costs. Last, the comorbid condition was measured by a self-reported single question asking whether respondents have more than four chronic conditions or not, which limited our ability to further examine whether certain comorbid conditions are more expensive to prevent/treat than the others.

Nevertheless, our findings have important policy implications. We advanced the literature in economics of mental health by documenting the association between severity of depressive symptoms and care costs across three settings, the cubic relationship between symptom severity and care expenditure, and such association among East Asian older adult population. Therefore, future studies can combine our baseline data with electronic health records and epidemiological data to project economic impacts and potential savings from investing in prevention and intervention strategies. Future studies can use our baseline data in economic models to evaluate the cost-effectiveness of new preventive/treatment interventions in Asia. Mild depression imposes a significant economic burden on society, with non-psychiatric healthcare being the major cost component. Investment in prevention and treatment of depression in primary care settings will likely be an effective cost-saving strategy.

## Conclusion

Depression is associated with significant economic impacts for healthcare and social care systems in an Asian city. The effect is larger among older adults with mild and moderately severe depressive symptoms. Older adults with similar symptom severity have very different care utilisation and expenditures: incremental associations between PHQ-9 score and care expenditures are not identical at different depression severities. Highest costs are seen in non-psychiatric healthcare settings, so screening of depressive symptoms should be included in these settings to provide proper diagnosis and treatment. Investment in prevention and treatment of depression in primary care settings would help reduce impacts of depression on the healthcare and long-term care system, facilitating their sustainability, especially given the rapidly ageing population in Asia.

## Data Availability

No additional unpublished data to share as the data collection is in progress.

## Supplementary material

For supplementary material accompanying this paper visit https://doi.org/10.1017/S2045796020001122. click here to view supplementary material

## References

[ref1] Basu A and Rathouz PJ (2005) Estimating marginal and incremental effects on health outcomes using flexible link and variance function models. Biostatistics (Oxford, England) 6, 93–109. 10.1093/biostatistics/kxh020 15618530

[ref2] Beecham J and Knapp M (1992) Costing psychiatric interventions. In Thornicroft G., Brewin C. R. and Wing J. (eds), Measuring Mental Health Needs. London: Gaskell/Royal College of Psychiatrists, pp. 163–183.

[ref3] Belotti F, Deb P, Manning WG and Norton EC (2015) Twopm: two-part models. Stata Journal 15, 3–20.

[ref4] Bock JO, Brettschneider C, Weyerer S, Werle J, Wagner M, Maier W, Scherer M, Kaduszkiewicz H, Wiese B, Moor L, Stein J, Riedel-Heller SG and Konig HH (2016) Excess health care costs of late-life depression: results of the AgeMooDe study. Journal of Affective Disorders 199, 139–147. 27104802 10.1016/j.jad.2016.04.008

[ref5] Bock JO, Hajek A, Weyerer S, Werle J, Wagner M, Maier W, Stark A, Kaduszkiewicz H, Wiese B, Moor L, Stein J, Riedel-Heller SG and König H-H (2017) The impact of depressive symptoms on healthcare costs in late life: longitudinal findings from the AgeMooDe study. The American Journal of Geriatric Psychiatry 25, 131–141. 27931772 10.1016/j.jagp.2016.10.011

[ref6] Chan CM, Chao T and Meng TG (2015) Chinese Conception of mental illness: a comparative culture analysis. Asian Journal of Pharmacy, Nursing and Medical Sciences 3, 1–7.

[ref7] Chao J, Li Y, Xu H, Yu Q, Wang Y and Liu P (2013) Health status and associated factors among the community-dwelling elderly in China. Archives of Gerontology and Geriatrics 56, 199–204. 23102740 10.1016/j.archger.2012.10.001

[ref8] Chen SL, Chiu HL, Xu BH, Ma Y, Jin T, Wu MH and Conwell Y (2010) Reliability and validity of the PHQ-9 for screening late-life depression in Chinese primary care. International Journal of Geriatric Psychiatry 25, 1127–1133. 20029795 10.1002/gps.2442

[ref9] Chin WY, Chan KTY, Lam CLK, Lam TP and Wan EYF (2015) Help-seeking intentions and subsequent 12-month mental health service use in Chinese primary care patients with depressive symptoms. BMJ Open 5, 1–10. 10.1136/bmjopen-2014-006730 PMC4316433 25631313

[ref10] Deb P and Norton EC (2018) Modeling health care expenditures and use. Annual Review of Public Health 39, 489–505. 10.1146/annurev-publhealth-040617-013517 29328879

[ref11] Garrido MM, Kane RL, Kaas M and Kane RA (2011) Use of mental health care by community-dwelling older adults. Journal of the American Geriatrics Society 59, 50–56. 21198461 10.1111/j.1532-5415.2010.03220.x PMC3050003

[ref12] Gratz KL (2001) Measurement of deliberate self-harm: preliminary data on the deliberate self-harm inventory. Journal of Psychopathology and Behavioral Assessment 23, 253–263.

[ref13] Guerra M, Prina AM, Ferri CP, Acosta D, Gallardo S, Huang Y, Jacob KS, Jimenez-Velazquez IZ, Rodriguez JJL, Liu Z, Salas A, Sosa AL, Williams JD, Uwakwe R and Prince M (2016) A comparative cross-cultural study of the prevalence of late life depression in low and middle income countries. Journal of Affective Disorders 190, 362–368. 26544620 10.1016/j.jad.2015.09.004 PMC4679114

[ref14] Knapp M and Wong G (2020) Economics and mental health: the current scenario. World Psychiatry 19, 3–14. 31922693 10.1002/wps.20692 PMC6953559

[ref15] König H, König HH and Konnopka A (2019) The excess costs of depression: a systematic review and meta-analysis. Epidemiology and Psychiatric Sciences 29, 1–16. 10.1017/S2045796019000180 PMC8061284 30947759

[ref16] Kroenke K, Spitzer R and Williams J (2000) A new measure of depression severity: the PHQ-9. Journal of General Internal Medicine 15, 78–78. 10.1046/j.1525-1497.2001.016009606.x PMC1495268 11556941

[ref17] Lam LCW, Wong CSM, Wang MJ, Chan WC, Chen EYH, Ng RMK, Hung SF, Cheung EFC, Sham PC, Chiu HFK, Lam M, Chang WC, Lee EHM, Chiang TP, Lau JTF, van Os J, Lewis G and Bebbington P (2015) Prevalence, psychosocial correlates and service utilization of depressive and anxiety disorders in Hong Kong: the Hong Kong Mental Morbidity Survey (HKMMS). Social Psychiatry and Psychiatric Epidemiology 50, 1379–1388. 25660760 10.1007/s00127-015-1014-5

[ref18] Lee DTS, Kleinman J and Kleinman A (2007) Rethinking depression: an ethnographic study of the experiences of depression among Chinese. Harvard Review of Psychiatry 15, 1–8. 17364968 10.1080/10673220601183915

[ref19] Ludvigsson M, Bernfort L, Marcusson J, Wressle E and Milberg A (2018) Direct costs of very Old persons with subsyndromal depression: a 5-year prospective study. The American Journal of Geriatric Psychiatry 26, 741–751. 29673895 10.1016/j.jagp.2018.03.007

[ref20] Lum TY, Parashuram S, Shippee TP, Wysocki A, Shippee ND, Homyak P, Kane RL and Williamson JB (2013) Diagnosed prevalence and health care expenditures of mental health disorders among dual eligible older people. The Gerontologist 53, 334–344. 23275518 10.1093/geront/gns163 PMC3888217

[ref21] McVittie C and Willock J (2006) “You can't fight windmills”: how older men do health, ill health, and masculinities. Qualitative Health Research 16, 788–801. 16760536 10.1177/1049732306288453

[ref22] Parker G, Gladstone G and Chee KT (2001) Depression in the planet's largest ethnic group: the Chinese. The American Journal of Psychiatry 158, 857–864. 11384889 10.1176/appi.ajp.158.6.857

[ref23] Porensky EK, Dew MA, Karp JF, Skidmore E, Rollman BL, Shear MK and Lenze EJ (2009) The burden of late-life generalized anxiety disorder: effects on disability, health-related quality of life, and healthcare utilization. The American Journal of Geriatric Psychiatry 17, 473–482. 19472438 10.1097/jgp.0b013e31819b87b2 PMC3408215

[ref24] Ramesh M and Holliday IAN (2001) The health care miracle in East and Southeast Asia: activist state provision in Hong Kong, Malaysia and Singapore. Journal of Social Policy 30, 637–651.

[ref25] Redondo-Sendino A, Guallar-Castillón P, Banegas JR and Rodríguez-Artalejo F (2006) Gender differences in the utilization of health-care services among the older adult population of Spain. BMC Public Health 6, 155. 16780576 10.1186/1471-2458-6-155 PMC1525176

[ref26] Schoevers RA, Smit F, Deeg DJH, Cuijpers P, Dekker J, Tilburg WV and Beekman ATF (2006) Prevention of late-life depression in primary care: do we know where to begin? The American Journal of Psychiatry 163, 1611–1621. 16946188 10.1176/ajp.2006.163.9.1611

[ref27] Sun WJ, Xu L, Chan WM, Lam TH and Schooling CM (2012) Depressive symptoms and suicide in 56,000 older Chinese: a Hong Kong cohort study. Social Psychiatry and Psychiatric Epidemiology 47, 505–514. 21384121 10.1007/s00127-011-0362-z PMC3304054

[ref28] The Hong Kong Council of Social Service (2015) Directory of Social Service Organizations in Hong Kong. Available at http://dss.hkcss.org.hk/contactus.php, Accessed Sep 10 2020.

[ref29] Vasiliadis HM, Dionne PA, Preville M, Gentil L, Berbiche D and Latimer E (2013) The excess healthcare costs associated with depression and anxiety in elderly living in the community. The American Journal of Geriatric Psychiatry 21, 536–548. 23567409 10.1016/j.jagp.2012.12.016

[ref30] Vegda K, Nie JX, Wang L, Tracy CS, Moineddin R and Upshur REG (2009) Trends in health services utilization, medication use, and health conditions among older adults: a 2-year retrospective chart review in a primary care practice. BMC Health Services Research 9, 217. 19948033 10.1186/1472-6963-9-217 PMC2791763

[ref31] Williams R (2012) Using the margins command to estimate and interpret adjusted predictions and marginal effects. Stata Journal 12, 308–331.

[ref32] Wong SY, Mercer SM, Leung J and Woo J (2009) The relationship between clinically relevant depressive symptoms and episodes and duration of all cause hospitalization in Southern Chinese elderly. Journal of Affective Disorders 113, 272–278. 18639936 10.1016/j.jad.2008.06.008

[ref33] Wong A, Nyenhuis D, Black SE, Law LSN, Lo ESK, Kwan PWL, Au L, Chan AYY, Wong LKS, Nasreddine Z and Mok V (2015) Montreal Cognitive assessment 5-Minute protocol is a brief, valid, reliable, and feasible cognitive screen for telephone administration. Stroke 46, 1059–1064. 25700290 10.1161/STROKEAHA.114.007253 PMC4373962

[ref34] World Health Organization (2017) Mental Health of Older Adults. Available at https://www.who.int/news-room/fact-sheets/detail/mental-health-of-older-adults, Accessed March 29 2020.

[ref35] World Health Organization (2015) World Report on Ageing and Health. Geneva: World Health Organization. Available at https://apps.who.int/iris/bitstream/handle/10665/186463/9789240694811_eng.pdf;jsessionid = 3CCE57D65100E0A6FF4DEDC883B4DCA2?sequence = 1.

[ref36] Yeung A, Fung F, Yu SC, Vorono S, Ly M, Wu S and Fava M (2008) Validation of the patient health questionnaire-9 for depression screening among Chinese Americans. Comprehensive Psychiatry 49, 211–217. 18243896 10.1016/j.comppsych.2006.06.002 PMC2268021

