# Supplementary material for: Health and social care service utilisation and associated expenditure among community-dwelling older adults with depressive symptoms
Source: Epidemiol Psychiatr Sci. 2021 Feb 2;30:e10. doi: 10.1017/S2045796020001122 (PMC8057460; doi:10.1017/S2045796020001122)
Supplement: Supplementary file 1 [file S2045796020001122sup001.docx]

**Supplementary Table 1**. Care categorisation and Unit costs (US dollar)

| **Care sectors** | **Unit cost** | **Source(s)** |
| --- | --- | --- |
| *Psychiatric services* |  |  |
| Psychiatric (inpatient) | $ 359 per day | HK Government Secretariat: Food and Health Bureau (Health Branch), 2019. Page 427. Cost per patient day (mentally ill) |
| Psychiatric (outpatient) | $ 212 per attendance | HK Government Secretariat: Food and Health Bureau (Health Branch), 2019. Page 427. Cost per psychiatric outreach attendance |
| Community-based Psychiatrists | $ 157 per outpatient attendance | HK Government Secretariat: Food and Health Bureau (Health Branch), 2019. Page 427. Cost per specialist outpatient attendance |
| Community-based Psychiatric nurses | $ 73 per visit | HK Government Secretariat: Food and Health Bureau (Health Branch), 2019. Page 427. Cost per outreach visit by community nurse |
| *Non-Psychiatric services* |  |  |
| General care (inpatient) | $ 632 per day | HK Government Secretariat: Food and Health Bureau (Health Branch), 2019. Page 427. Cost per patient day (general) |
| General care (outpatient) | $ 128 per attendance | HK Government Secretariat: Food and Health Bureau (Health Branch), 2019. Page 427. The average cost of A&E attendance ($ 195) and general outpatient attendance ($60). |
| Community-based General Practitioner visits | $ 60 per attendance | HK Government Secretariat: Food and Health Bureau (Health Branch), 2019. Page 427. Cost per general outpatient attendance |
| Community-based nurses | $ 73 per visit | HK Government Secretariat: Food and Health Bureau (Health Branch), 2019. Page 427. Cost per outreach visit by community nurse |
| *Rehabilitation* |  |  |
| Geriatric day hospital | $ 286 per day attendance | HK Government Secretariat: Food and Health Bureau (Health Branch), 2019. Page 427. Cost per geriatric day attendance |
| Occupational therapist consultation | $ 29 per hour | HK Social Welfare Department (2018). The middle point of monthly salary of an Occupational Therapist II is $ 4,682. The hourly rate is equal to monthly salary divided by 160 estimated working hours per month. |
| *Social care providers* |  |  |
| Social workers | $ 41 per hour | HK Social Welfare Department (2018). The mid-point of monthly salary of an Assistant Social Work Officer is $ 6,491. The hourly rate is equal to the monthly salary divided by 160 estimated working hours per month. |
| Clinical psychologist | $ 63 per hour | HK Social Welfare Department (2018). The mid-point of monthly salary of a Clinical Psychologist is $ 10,010. The hourly rate is equal to the monthly salary divided by 160 estimated working hours per month. |
| Personal care helper | $ 14 per hour | HK Social Welfare Department (2018). The mid-point of monthly salary of a Personal Care Worker is $ 2,280. The hourly rate is equal to the monthly salary divided by 160 estimated working hours per month. |

Reference: HK Government Secretariat: Food and Health Bureau (Health Branch), 2019. Head 140. Retrieved from https://www.budget.gov.hk/2019/eng/pdf/head140.pdf. Accessed May 30, 2020. HK Social Welfare Department (2018). Salary Scale of Common Posts in the Non-governmental Organisations w.e.f. 1.4.2018. Retrieved from https://www.swd.gov.hk/storage/asset/section/728/en/Salary_Scales_of_Common_Posts_w.e.f._01.04.2018.pdf. Accessed May 30, 2020. U.S. Department of Treasury (2018) Treasury reporting rates of exchange (h<ttps://fiscal.treasury.gov/files/reports-statements/treasury-reporting-rates-exchange/itin-12-31-2018.pdf)>. Accessed 29 May 2020.

**Supplementary Table 2**. Sensitivity analysis: Results of the two-part model analysis on care expenditures without a previous diagnosis of depression/anxiety (N=2,707)

|  | **Health care** | | **Rehabilitation** | | **Social care** | | **Total** | |
| --- | --- | --- | --- | --- | --- | --- | --- | --- |
|  | Logit | GLM | Logit | GLM | Logit | GLM | Logit | GLM |
|  | OR | Estimate | OR | Estimate | OR | Estimate | OR | Estimate |
|  | (S.E.) | (S.E.) | (S.E.) | (S.E.) | (S.E.) | (S.E.) | (S.E.) | (S.E.) |
| *Severity level (ref.: non-depressed)* |  |  |  |  |  |  |  |  |
| *Mild* | 1.59*** | 0.92*** | 2.02** | 0.39 | 0.76* | 0.53 | 1.51** | 0.95*** |
|  | (0.19) | (0.15) | (0.46) | (0.32) | (0.1) | (0.27) | (0.19) | (0.14) |
| Moderate | 1.75** | 0.57*** | 1.90* | -0.58 | 1.71** | -0.39 | 1.63** | 0.54*** |
|  | (0.31) | (0.15) | (0.57) | (0.37) | (0.29) | (0.28) | (0.31) | (0.15) |
| Moderately Severe | 4.19** | 1.25*** | 3.16** | -0.35 | 2.31** | -0.06 | 3.60** | 1.22*** |
|  | (1.81) | (0.32) | (1.27) | (0.48) | (0.64) | (0.32) | (1.58) | (0.31) |
|  | **Psychiatric care** | | **Non-Psychiatric care** | |  | | | |
|  | Logit | GLM | Logit |  |  |  |  |  |
|  | OR | Estimate | OR | Estimate |  |  |  |  |
|  | (S.E.) | (S.E.) | (S.E.) | (S.E.) |  |  |  |  |
| *Severity level (ref.: non-depressed)* |  |  |  |  |  |  |  |  |
| *Mild* | 1.92*** | 0.02 | 1.45** | 0.94*** |  |  |  |  |
|  | (0.36) | (0.17) | (0.17) | (0.15) |  |  |  |  |
| Moderate | 4.27*** | 0.33 | 1.48* | 0.50** |  |  |  |  |
|  | (0.9) | (0.19) | (0.25) | (0.16) |  |  |  |  |
| Moderately Severe | 6.34*** | 0.08 | 3.41** | 1.24*** |  |  |  |  |
|  | (1.79) | (0.21) | (1.29) | (0.34) |  |  |  |  |

Notes: Results controlled for gender, age, marital status, education, poverty status, living alone, Montreal Cognitive Assessment, and chronic diseases. OR: Odds Ratio; SE: Standard Error; β: Coefficient; GLM: Generalized Linear Model.

**Supplementary Table 3**. Results of the two-part model analysis on care expenditures using PHQ-9 score as a continuous variable (N=2,707)

|  | Health care | | Rehabilitation | | Social care | | Overall | | Psychiatric care | | Nonpsychiatric care | |
| --- | --- | --- | --- | --- | --- | --- | --- | --- | --- | --- | --- | --- |
|  | Logit | GLM | Logit | GLM | Logit | GLM | Logit | GLM | Logit | GLM | Logit | GLM |
|  | OR (SE) | β (SE) | OR (SE) | β (SE) | OR (SE) | β (SE) | OR (SE) | β (SE) | OR (SE) | β (SE) | OR (SE) | β (SE) |
| PHQ-9 Score | 1.24** | 0.31*** | 1.06** | -0.05 | 1.05** | -0.02 | 1.18* | 0.34*** | 0.99 | 0.07 | 1.23** | 0.32*** |
|  | *(-0.1)* | *(-0.08)* | *(-0.02)* | *(-0.03)* | *(-0.01)* | *(-0.02)* | *(-0.09)* | *(-0.08)* | *(-0.11)* | *(-0.08)* | *(-0.09)* | *(-0.08)* |
| PHQ-9 Score^2^ | 0.98 | -0.02* | - | - | - | - | 0.99 | -0.03** | 1.02 | 0 | 0.98* | -0.02* |
|  | *(-0.01)* | *(-0.01)* | *(-)* | *(-)* | *(-)* | *(-)* | *(-0.01)* | *(-0.01)* | *(-0.01)* | *(-0.01)* | *(-0.01)* | *(-0.01)* |
| PHQ-9 Score^3^ | 1.001 | 0 | - | - | - | - | 1 | 0.001 | 1 | 0 | 1.001 | 0 |
|  | *(0)* | *(0)* |  |  |  |  | *(0)* | *(0)* | *(0)* | *(0)* | *(0)* | *(0)* |
| *Control variables* |  |  |  |  |  |  |  |  |  |  |  |  |
| Female | 0.93 | -0.92*** | 0.94 | -0.18 | 0.84 | -0.61* | 0.89 | -0.89*** | 0.85 | 0.30* | 1.04 | -0.96*** |
|  | *(-0.13)* | *(-0.19)* | *(-0.21)* | *(-0.29)* | *(-0.12)* | *(-0.3)* | *(-0.13)* | *(-0.18)* | *(-0.16)* | *(-0.14)* | *(-0.14)* | *(-0.19)* |
| Married | 0.91 | -0.24 | 1.03 | 0.09 | 1.17 | -0.17 | 0.92 | -0.24 | 0.68* | 0.05 | 0.96 | -0.22 |
|  | *(-0.13)* | *(-0.18)* | *(-0.23)* | *(-0.32)* | *(-0.19)* | *(-0.25)* | *(-0.13)* | *(-0.18)* | *(-0.13)* | *(-0.13)* | *(-0.13)* | *(-0.18)* |
| Age | 1.026** | 0.03*** | 1 | -0.02 | 1.01 | -0.01 | 1.04*** | 0.03** | 0.95*** | 0 | 1.04*** | 0.04*** |
|  | *(-0.01)* | *(-0.01)* | *(-0.01)* | *(-0.02)* | *(0)* | *(-0.01)* | *(-0.01)* | *(-0.01)* | *(-0.01)* | *(-0.01)* | *(-0.01)* | *(-0.01)* |
| Years of education | 1.02 | 0 | 0.99 | -0.02 | 0.98 | 0.04 | 1.01 | 0 | 1.02 | 0.01 | 1.02 | 0 |
|  | *(-0.01)* | *(-0.02)* | *(-0.02)* | *(-0.03)* | *(-0.01)* | *(-0.04)* | *(-0.01)* | *(-0.02)* | *(-0.02)* | *(-0.02)* | *(-0.01)* | *(-0.02)* |
| Living alone | 0.97 | -0.27 | 1.07 | 0.08 | 2.33*** | -0.71* | 1.18 | -0.29 | 0.94 | 0.41* | 0.92 | -0.27 |
|  | *(-0.14)* | *(-0.21)* | *(-0.22)* | *(-0.3)* | *(-0.37)* | *(-0.26)* | *(-0.18)* | *(-0.21)* | *(-0.18)* | *(-0.16)* | *(-0.12)* | *(-0.21)* |
| Poverty status: Welfare recipients | 1.27 | 0.12 | 1.07 | 0.76** | 1.60*** | 1.52*** | 1.35* | 0.19 | 1.31 | 0.25 | 1.15 | 0.11 |
|  | *(-0.17)* | *(-0.17)* | *(-0.2)* | *(-0.28)* | *(-0.2)* | *(-0.29)* | *(-0.2)* | *(-0.17)* | *(-0.22)* | *(-0.16)* | *(-0.15)* | *(-0.18)* |
| MoCA | 1 | -0.01 | 0.99 | 0.06 | 1 | -0.003 | 1 | -0.01 | 1.02 | -0.02 | 0.99 | -0.01 |
|  | *(-0.02)* | *(-0.02)* | *(-0.02)* | *(-0.03)* | *(-0.02)* | *(-0.03)* | *(-0.02)* | *(-0.02)* | *(-0.02)* | *(-0.02)* | *(-0.01)* | *(-0.02)* |
| Previous diagnosis of depression/anxiety | 1.54 | 0.25 | 1.4 | -0.3 | 1.08 | 0.03 | 1.46 | 0.24 | 8.74*** | -0.27* | 0.94 | 0.22 |
|  | *(-0.35)* | *(-0.18)* | *(-0.38)* | *(-0.33)* | *(-0.23)* | *(-0.31)* | *(-0.34)* | *(-0.19)* | *(-1.59)* | *(-0.13)* | *(-0.18)* | *(-0.21)* |
| With more than 4 chronic diseases | 2.14*** | -0.01 | 2.76*** | 0.25 | 1.26 | 0.54 | 2.41*** | 0.08 | 0.85 | -0.37** | 2.33*** | -0.01 |
|  | *(-0.47)* | *(-0.15)* | *(-0.59)* | *(-0.32)* | *(-0.22)* | *(-0.42)* | *(-0.59)* | *(-0.15)* | *(-0.19)* | *(-0.14)* | *(-0.5)* | *(-0.16)* |
| Constant | 0.37 | 5.79*** | 0.05* | 8.46*** | 0.02 | 7.52*** | 0.2 | 6.20*** | 1.06 | 6.81*** | 0.19* | 5.62*** |
|  | *(-0.31)* | *(-0.92)* | *(-0.07)* | *(-1.94)* | *(-0.02)* | *(-1.23)* | *(-0.17)* | *(-0.91)* | *(-1.3)* | *(-0.83)* | *(-0.16)* | *(-0.98)* |

Notes: MoCA: Montreal Cognitive Assessment; OR: Odds Ratio; β: Coefficient; GLM: Generalized Linear Model; SE: Standard Error; Standard errors in parentheses. PHQ-9: Patient Health Questionnaire. * p<0.05; ** p<0.01; *** p<0.001.
